# Overexpression of the *Panax* *ginseng* *CYP703* Alters Cutin Composition of Reproductive Tissues in Arabidopsis

**DOI:** 10.3390/plants11030383

**Published:** 2022-01-30

**Authors:** Jihyun Kim, Jeniffer Silva, Chanwoo Park, Younghun Kim, Nayeon Park, Johan Sukweenadhi, Junping Yu, Jianxin Shi, Dabing Zhang, Keun Ki Kim, Hong-Joo Son, Hyeon Cheal Park, Chang-Oh Hong, Kwang Min Lee, Yu-Jin Kim

**Affiliations:** 1 Department of Life Science and Environmental Biochemistry, Life and Industry Convergence Research Institute, Pusan National University, Miryang 50463, Korea; jihyun0_0@pusan.ac.kr (J.K.); musgdmu8869@naver.com (C.P.); edward69543@naver.com (Y.K.); xosk126@naver.com (N.P.); kkkim@pusan.ac.kr (K.K.K.); shjoo@pusan.ac.kr (H.-J.S.); hcpark@pusan.ac.kr (H.C.P.); soilchem@pusan.ac.kr (C.-O.H.); leekm@pusan.ac.kr (K.M.L.); 2 Department of Research and Development, The Bridge Biofoundry, Ciudad de Saber, Clayton 0843-03081, Panama; jeniffersilva.yat@gmail.com; 3 Department of Plant Biotechnology, Faculty of Biotechnology, Universitas Surabaya, Raya Kalirungkut, Kalirungkut, Surabaya 60294, East Java, Indonesia; sukweenadhi@gmail.com; 4 Key Laboratory of Biotechnology Shaanxi Province, College of Life Sciences, Chinese Education Ministry’s Key Laboratory of Western Resources and Modern Biotechnology, Northwest University, Xi’an 710069, China; yujunping521@sina.com; 5 Joint International Research Laboratory of Metabolic and Developmental Sciences, School of Life Sciences and Biotechnology, Shanghai Jiao Tong University, Shanghai 200240, China; jianxin.shi@sjtu.edu.cn (J.S.); zhangdb@sjtu.edu.cn (D.Z.)

**Keywords:** cytochrome P450, reproductive tissues, *PgCYP703A4*, fatty acid, reproduction, *Panax ginseng*

## Abstract

Cytochrome P450 (CYP) catalyzes a wide variety of monooxygenation reactions in plant primary and secondary metabolisms. Land plants contain CYP703, belonging to the CYP71 clan, which catalyzes the biochemical pathway of fatty acid hydroxylation, especially in male reproductive tissues. Korean/Asian ginseng (*Panax ginseng* Meyer) has been regarded as one of important medicinal plant for a long time, however the molecular mechanism is less known on its development. In this study, we identified and characterized a *CYP703A* gene in *P. ginseng* (*PgCYP703A4*), regarding reproductive development. *PgCYP703A4* shared a high-sequence identity (81–83%) with predicted amino acid as CYP703 in *Dancus carota*, *Pistacia vera*, and *Camellia sinensis* as well as 76% of amino acid sequence identity with reported *CYP703* in *Arabidopsis thaliana* and 75% with *Oryza sativa*. Amino acid alignment and phylogenetic comparison of *P. ginseng* with higher plants and known *A. thaliana* members clearly distinguish the CYP703 members, each containing the AATDTS oxygen binding motif and PERH as a clade signature. The expression of *PgCYP704B1* was only detected in *P. ginseng* flower buds, particularly in meiotic cells and the tapetum layer of developing anther, indicating the conserved role on male reproduction with At- and Os- CYP703. To acquire the clue of function, we transformed the *PgCYP703A4* in *A. thaliana*. Independent overexpressing lines (*PgCYP703A4*ox) increased silique size and seed number, and altered the contents of fatty acids composition of cutin monomer in the siliques. Our results indicate that *PgCYP703A4* is involved in fatty acid hydroxylation which affects cutin production and fruit size.

## 1. Introduction

The cytochrome P450 (CYP) superfamily of enzymes, which catalyze diverse substrates through oxygenation and hydroxylation reactions, are found in all organisms [1]. Plant CYPs are involved in a variety of biochemical pathways that produce primary and secondary metabolites, which constitute one of the largest families of enzymes in higher plants [2]. The diversification of land plant CYP families emerges during flowering plant evolution and specializes plant species with unique reactions [3]. The identification of CYP genes aids in understanding the evolution of various groups of enzymes and their conserved and diversified functions.

The *CYP703* gene family was found across the land plant taxa, suggesting that it encodes an essential function [4]. Land plants developed specialized cell layer to adapt to the environment, including cutin synthesis. As cutin cover in most plant organ of land plant, male reproductive organ of flowering plants is covered with a hydrophobic polymer barrier derived from fatty acids. The production of functional gametophytes leads to a successful proliferation in flowering plants [5]. Male reproduction is a complex and highly coordinated biological process that includes the development of the male reproductive organ: the stamen that contains the microspores and pollen. The study of male reproduction is important not only for increasing crop yield but for producing improved crops, such as superhybrid plants, through the use of male sterile lines [6]. 

The CYP703 family is involved in the in-chain hydroxylation of mid-chain fatty acids, which is essential for the biopolymers of pollen exine, the outer wall of a pollen grain [7,8], and cutin, one of the most abundant lipid polymers and an important adaptive trait of plants to their terrestrial environment [9]. *A. thaliana* CYP703A2 (AtCYP703A2) catalyzes the conversion of medium-chain saturated fatty acids to the corresponding monohydroxylated fatty acids, with a preferential hydroxylation of lauric acid at the C-7 position [8]. In comparison, *Oryza sativa* CYP703A3 (OsCYP703A3) can only catalyze in-chain hydroxylation of lauric acid (C12), preferentially at position 7 [7]. The *A. thaliana* mutant, *cyp703a2*, exhibits impaired pollen development and a partial male sterile phenotype due to the lack of an exine [8]. *O. sativa* CYP703A3 resulted in a complete male sterility with an abnormal anther epidermis, as well as defective pollen exine, indicating that the diversified function of CYP703A during evolution [7]. Both participated in a conserved pathway of in-chain hydroxylation of lauric acid that is required for male reproductive development. Some male-specific gene sequences that determine sex, where CYP703 was found, have been identified in the *Phoenix* tree which belongs to dioecious species. [10]. Further studies in other plants will help elucidate the diversified CYP703 function of fatty acid hydroxylation in plant reproductive development.

*Panax ginseng* is a perennial herb that has been cultivated for its highly valued root for medicinal purposes [11]. *P. ginseng* typically starts its reproduction at the third year of growth [12,13]. Attempts to increase the yield of *P. ginseng* and ginsenosides have been conducted by developing *P. ginseng* hybrids, and although they display heterosis, F1 hybrid plants exhibited male sterility that derived from pollen defects at the young microspore stage [13]. We previously studied and described the morphogenesis of the anther and carpel at a cytological level to understand and specify the reproductive developmental phases of *P. ginseng* [12,14] and identified the gene expression of *PgCYP703* in the anther tapetum layer [12]. Despite the importance of *P. ginseng* reproductive development, studies on functional gene analysis and molecular regulation remain scarce. In the studies presented here, we isolated and cloned the *CYP703A* gene from *P. ginseng*, *PgCYP703A*, which is highly expressed in flower buds during anther development. Surprisingly, in *A. thaliana*, overexpression of *PgCYP704B1* resulted in the enhanced in silique length, in terms of fruit size, which is potentially caused by the alteration of saturated fatty acids and hydroxy fatty acids in siliques.

## 2. Material and Methods

### 2.1. Plant Materials and Growth Conditions

The ginseng (*P. ginseng* Mayer) plant organs (root body, stem, leaf, flower bud, and fruit) were obtained from hydroponically cultured ginseng. Columbia ecotype (CS60000) of *A. thaliana* was used for gene overexpression. Sterilized seeds were sown on half-strength Murashige and Skoog medium (Duchefa Biochemie) containing 1% sucrose, 0.8% (*w*/*v*) agar, and pH 5.7. Three-day-old cold-treated seeds were germinated under long-day photoperiods of 16-h light/8-h dark at 23 °C. The transformants were screened on hygromycin (50-μg/mL)-selective medium plates. Ten-day-old seedlings were then transplanted to soil and cultivated for five weeks under the same light/dark conditions [15].

### 2.2. Identification of PgCYP703A4 Gene and Sequence Analysis

To obtain a coding sequence (CDS) of the *PgCYP703A4* gene, homologous sequences of *CYP703* was obtained based on *A. thaliana* sequence by homology-based PCR from *P. ginseng* flower cDNA. A complete genomic DNA sequence was obtained and analyzed from the database of the *P. ginseng* genome (http://ginsengdb.snu.ac.kr, accessed on 20 January 2022), and the putative ORF sequence was verified by sequencing after subcloning. 

The predicted amino acid sequence of *PgCYP703A4* was used to search for homologous proteins via National Center for Biotechnology Information-Basic Local Alignment Search Tool (NCBI-BLASTX, http://www.ncbi.nlm.nih.gov/BLAST/, accessed on 18 January 2022). Sequence alignment was conducted using Clustal X V1.83, and a neighbor-joining tree was constructed using the MEGA4 software V.4.0.1, with the reliability of each node established by the bootstrap method. The subcellular localization for the N-terminus was predicted by PSORTdb (http://www.psort.org/psortb/, accessed on 18 January 2022) [16], and the hydropathy value was calculated using the previously described method [17]. The 1000-bp sequence upstream of the ATG-coding site in the genomic DNA sequence of *PgCYP703* were used as promoters to predict Cis-acting elements by New PLACE (http://www.dna.affrc.go.jp/PLACE/?action=newplace, accessed on 20 December 2021) [18].

### 2.3. Vector Construction and A. thaliana Transformation

The full-length *CYP703* gene was amplified from *P. ginseng* flower cDNA and cloned into the *Sal*I and *Spe*I sites of the pCAMBIA1390 vector containing the Cauliflower Mosaic Virus 35S promoter and yellow fluorescent protein. After nucleotide sequence verification, *A. thaliana* transformation was conducted using *Agrobacterium tumefaciens* C58C1 (pMP90) [19]. The insertion of transgenes into the transformants was confirmed via polymerase chain reaction (PCR). Heterozygous plants with a 3:1 segregation ratio on antibiotic plates which indicate the single gene insertion, were selected for additional analyses. Among the several T2 independent lines, two lines were selected for further statistical and metabolite analyses.

### 2.4. Gene Expression Analysis

Total RNA extraction from frozen samples was performed using the RNeasy Mini Kit (Qiagen, Valencia, CA, USA.), where 1-μg of the total RNA was used as a reverse transcription template. For qRT-PCR, 100-ng cDNA in a 10-μL reaction volume and SYBR^®^ Green Sensimix Plus Master Mix (Quantace, Watford, England) were used. Specific primers for *PgCYP703A4* (F-5′-CTACGGGTGCAATGATGTTG-3′ and R-5′- TGCATGGAAAACGACTCAAG-3′) and a constitutively expressed *P. ginseng actin* gene (forward, 5′-AGAGATTCCGCTGTCCAGAA-3′ and reverse, 5′-ATCAGCGATACCAGGGAACA-3′) or *A. thaliana actin* gene (forward, 5′-GTGTGTCTTGTCTTATCTGGTTCG-3′ and reverse 5′-AATAGCTGCATTGTCACCCGATACT-3′) were used as an internal reference. qRT-PCR was conducted using a CFX Connect Real-Time PCR Detection System (BIO-RAD, Hercules, CA, USA) with the following program: 30 s at 95 °C, followed by 40 cycles of 95 °C for 3 s and 60 °C for 20 s. The threshold cycle (Ct) reflects the number of cycles where the fluorescence intensity at the original exponential stage of PCR amplification was significantly greater than the background fluorescence. To determine the relative fold differences in template abundance for each sample, the Ct value for *PgCYP703A4* was normalized to the Ct value for β-actin and calculated relative to a calibrator using the formula 2^−ΔΔCt^. Each qRT-PCR was technically repeated at least three times. Spatial expression of PgCYP703 transcript in ginseng anther was analyzed by In situ hybridization, as reported previously [12].

### 2.5. Histological Analysis

Semi-thin sectioning was performed using anthers of mature flowers from 4-week-old plants. After fixing in FAA, the samples were dehydrated with an ethanol gradient (70%, 80%, 90%, and 100%) allowing 30 min for each step. Samples were then embedded in KULZER Technovit 7100 cold polymerizing resin by pre-infiltration, infiltration, and embedding at 45 °C, according to the previously described methods [20]. Samples were sectioned to a thickness of 4 µm in an Ultratome III ultramicrotome (LBK) and stained with 0.25% toluidine blue O (Chroma Gesellshaft Shaud). Bright-field photographs of the anther and silique sections were obtained using a Nikon ECLIPSE 80i microscope.

Scanning electron microscopy (SEM) was employed to analyze mature anthers that were fixed in FAA and dehydrated using 20–100% ethanol (10% increments) allowing 3 min for each step. The samples were then dried at the critical point temperature (Leica EM CPD300). A 5-nm-thick Aurum coating was paced on the samples with a Leica EM SCD050 ion sputter. The Aurum-coated samples were observed using a Hitachi S3400N SEM.

### 2.6. Analysis of Silique Fatty Acids

Cutin from the siliques of 5-week-old plants were examined as previously described [15,21]. Dried siliques (10–20 mg) were extracted in 2 mL of chloroform and spiked with 10 μg of tetracosane (Fluka) as the internal standard. Solvent was evaporated under a light stream of nitrogen, and the compounds containing free hydroxyl and carboxyl groups were transformed to trimethysilyl ethers and esters using 20-μL bis-(N, N-trimethysilyl)-trifluoroacetamine (Sigma-Aldrich, St. Louis, MO, USA) in 20-μL pyridine for 40 min at 70 °C. The monomers were identified from their electron ionization–mass spectrometry spectra (70 eV, *m*/*z* 50 to 700) after GC separation (column 30 mm × 0.32 mm × 0.1 μm film thickness [DB-1; J&W Scientific]). Gas chromatography–mass spectrometry (GC-MS) (Agilent gas chromatograph coupled with an Agilent 5973N quadrupole mass selective detector) and gas chromatography–flame ionization detection (GC-FID) (Agilent 6890 gas chromatograph) analyses were conducted. The means of three independent replicates were statistically analyzed and compared with control (* *p* < 0.05) using Student’s *t*-test. 

## 3. Results

### 3.1. Identification of CYP703 Gene in P. ginseng

**Figure 1:**
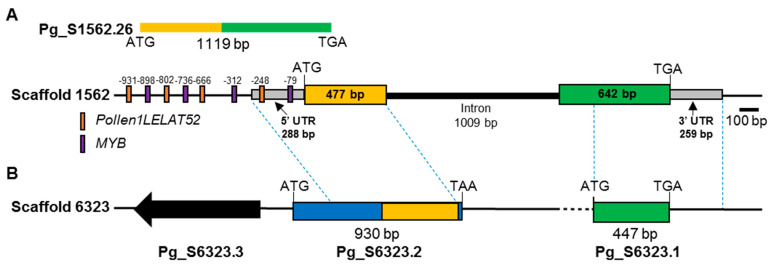
Analysis of gene and promoter structure of PgCYP703A4 and its pseudogene. Genomic sequence of scaffolds containing PgCYP703A4 and similar sequences were identified from the P. *ginseng* genome database (http://ginsengdb.snu.ac.kr/ accessed on 20 January 2022). (**A**) PgCYP703A4 gene was confimed as Pg_S1562.26 CDS, which encoded on Scaffold 1562. The coding regions (orange and green boxes) are interrupted by 1009 base pair (bp) intron. The upstream 1000-bp region from the translation start site has four POLLEN1LELAT52 binding-predicted sites and four MYBCORE binding-predicted sites. (**B**) A similar sequence structure was identified from Pg_scaffold6323 encoding two CDSs, assumed to be pseudogenes. The transcript was separated into two partial CDS sequences (Pg_S6323.2 and Pg_S6323.1). Dashed line indicates closed sequences between two scaffolds.

CYP families often have many paralogs, but the CYP703 family was reported to be a single-gene-member family [8]. Analysis of the *P. ginseng* genome scaffold and CDS revealed that *P. ginseng* contains two scaffold sequences with high similarity to CYP703. Among the two scaffolds, scaffold 1562 contained a full-length CDS of CYP703 (Pg_S1562.26), which contained a two-exons and one-intron structure (Figure 1), similar to the *A. thaliana* gene structure. On the contrary, two CDS sequences, S6323.1 and S6323.2, present in scaffold 6323 were partial sequences of CYP703. Therefore, we concluded that the *P. ginseng* genome encodes just one PgCYP703 member, whereas the others are nonfunctional genes. The recent genome duplications of the *P. ginseng* genome [22] might explain the presence of two genes that can duplicated, in which one of the gene has retained the original sequence and function, whereas the other became a pseudogene. Similarly, CYP703A was noted as a single functional sequence in the poplar genome (*Populus trichocarpa*) [8].

A putative ORF sequence, which had a length of 1119 bp and encoded 372 amino acids (Figure 1), was verified by sequencing, and an NCBI-BLAST search displayed the conserved superfamily CYP. There are three functionally reported genes: two CYP703A members registered in the Plant P450 Database (http://erda.dk/public/vgrid/PlantP450/, accessed on 10 December 2021) (CYP703A1 from a *Petunia hybrida* [23] and AtCYP703A2 [8]) and *O. sativa* CYP703A3 [7]. We named the CYP703 gene identified in *P. ginseng* as *PgCYP703A4*.

### 3.2. Sequence Alignment and Phylogenetic Analysis

**Figure 2:**
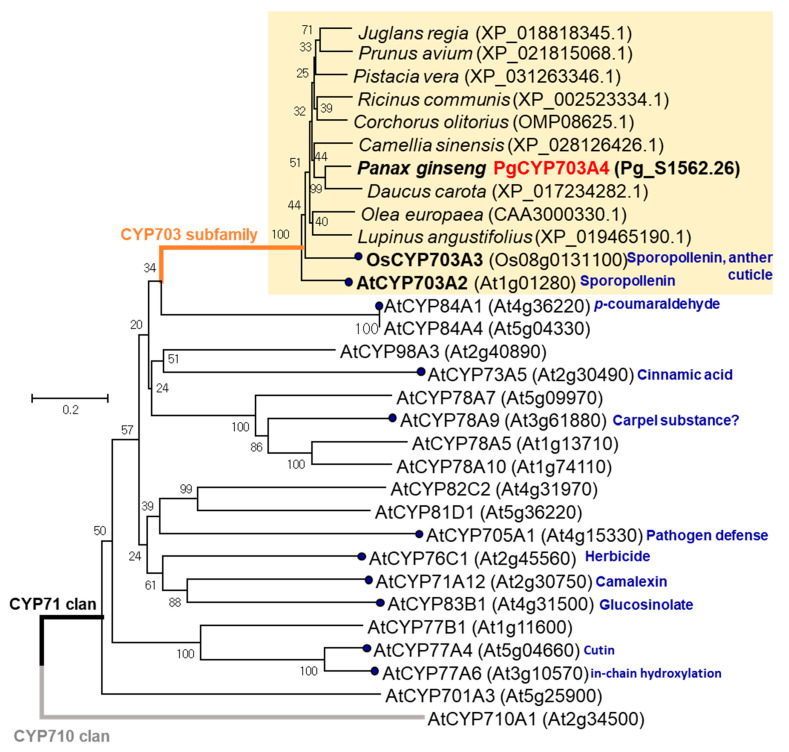
Phylogenetic analysis of PgCYP703. Neighbor-joining method analysis was conducted using the full-length amino acid sequences of PgCYP703A4 and closely related CYP703 subfamily members (Figure S1), in addition to the A. thaliana members of the CYP71 clan and outgroup of the CYP710 clan. The scale bar shows 0.2 amino acid substitutions per site. The reported CYP703A genes are distinguished by bold font with black dots. CYP703 subfamilies of the CYP71 clan are indicated by the yellow box. The functionally reported genes are indicated by circles and brief role in the right side. In figure, ‘At’ means *Arabidopsis thaliana*’s protein and ‘Os’ means *Oryza sativa*’s protein, and other plants are presented with full scientific name. NCBI accession numbers for other species and annotation numbers for *P. ginseng*, *A. thalina* and *O. sativa* species are indicated inside bracket.

CYP703 enzymes belong to the CYP71 clan, which includes the diverse families of P450 in plants [4,7]. To obtain information about the potential functions and evolutionary roles of *PgCYP703A4*, the full-length protein was used as a query to search for homologs in NCBI databases and the The *A. thaliana* Information Resource using NCBI-BLASTX. Highly similar homologs of PgCYP703A4 were detected in various dicot plant species whose genome sequences were available, although functional studies were limited. To see similarity of amino acids in various plant species, we selected the 10 closest sequences, and representative CYP71 members from *A.* *thaliana* were used to create a phylogenetic tree (Figure 2). Based on our phylogenetic comparison, *PgCYP703A4* was placed in subfamily CYP703, separate from the other subfamilies of the CYP71 clan; CYP98, CYP73, CYP78, CYP84, CYP82, CYP81, CYP76, CYP61, CYP83, CYP705, and CYP701. CYP78 members hydoxylate short-chain fatty acids [24], CYP73 family members hydroxylate cinnamic acid [25], and CYP84 members are involved in lignin and flavonoid synthesis [26], thus indicating CYP71 clan subfamilies are not specific, rather showed various functions on metabolites.

Multiple sequence alignment (Figure S1) revealed that the CYP703 proteins and PgCYP703A4 contain the conserved domains of the axial ligand for heme, the I-helix involved in oxygen binding, the Arg of the “PERF” consensus, and the E-R-R of the K-helix consensus (KETLR). Of these domains, only the cysteine of the heme-binding domain and the E-R-R triad are conserved in all plant cytochrome P450 sequences [27,28]. CYP703 members have a Phe–His substitution in the P(E)R(F) domain of CYP, known as a clade signature of the CYP703 family [28]. In addition, CYP703 members have an A-A-T-D-T-S motif (Figure S1) in the A/G-G-X-E/D-T-T/S domain, which is involved in oxygen binding and activation [28]. Of the subfamilies in the CYP71 clan, substitution of A for the second G is unique to the CYP703 subfamily. 

Plant P450s are usually bound to membranes, anchoring to the cytoplasmic surface of the endoplasmic reticulum (ER) through a short hydrophobic segment of their N-terminus [29] The predicted transit peptide of PgCYP703A4 (indicated arrow in Figure S1) was shown to be positioned at its N-terminus with a cytoplasmic location [16] targeting ER with 69% certainty, predicted by PSORT (Prediction of Protein Localization Sites, version 6.4) Prediction program. Fatty acids are hydroxylated in the ER of plant cells through members of the CYP family [8,30,31]. The PgCYP703 hydrophobicity profile and those of its closest homologs indicated that both the N- and C-terminal regions, as well as the CYP703 motifs, are highly conserved (Figure S2). 

### 3.3. Gene Expression Analysis of PgCYP703A4

**Figure 3:**
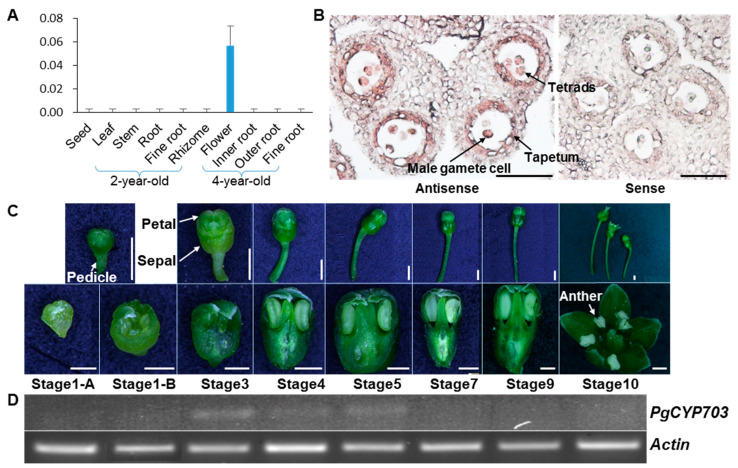
Tissue expression analysis of *PgCYP703A4* in *P. ginseng*. (**A**) Quantitative expression analysis of *PgCYP703A4* in various tissues at different age of *P. ginseng* plant. The expression levels were analyzed via realtime PCR, and *PgActin* served as the control. Values indicate mean of three technical replicates ± SE. (**B**) In situ hybridization analysis of *PgCYP703A4* in *P. ginseng* anther at stage 4 showing its expression (dark pink) in tapetal cells and microspores. Right image shows the anther with hybridized *PgCYP703A4* sense probe, as control. Scale bars indicate 500 μm. (**C**) Flower tissues of the *P. ginseng* at anther developmental stages [14] were used for RT-PCR. The scale bars of uppder photo indicate 1 mm, and lower photo indicate 500 μm. (**D**) RT-PCR gel images of PgCYP703A4 at seven stages of *P. ginseng* flowers show that *PgCYP703A4* are expressed only during Stage 3 to 5. *PgActin* served as a control.

To verify the conserved function of *PgCYP703A4* in the male reproductive organ, as has been described for *A. thaliana* [8] and *O. sativa* [7], we conducted *PgCYP704B1* expression analysis via qRT-PCR using *P. ginseng* tissues, such as the see, root, stem, leaf, flower buds, and fruit at different age. *PgCYP703A4* was specifically expressed in flower buds (Figure 3A). 

In situ hybridization experiments revealed that *PgCYP703A4* was expressed in the tapetal cell layer and tetrad cells (meiocytes after the second meiosis) in the anther (Figure 3B). The tapetum, the innermost layer of anther wall, plays a crucial role in pollen development by nursing and releasing the microspore [32]. During the developmental stage of anther, the *PgCYP703A4* expression was observed in meiosis to form young microspores (Figure 3C,D) [13]. 

To illuminate the regulation of gene expression, the 1000-bp upstream region from the coding sequence of PgCYP703A4 was analyzed for cis-elements (Figure 1). The promoters contained various elements. Among them, we noted POLLEN1LELAT52 and MYBCORE, known as regulatory factors that are related to pollen and early pollen development, respectively [33,34]. These results indicated that MYBCORE and POLLEN1LELAT52 might bind to the promoters of *PgCYP703*, which leads to their specific expression during anther development. 

### 3.4. Phenotype Analysis of PgCYP3A4 Overexpressing A. thaliana

**Figure 4:**
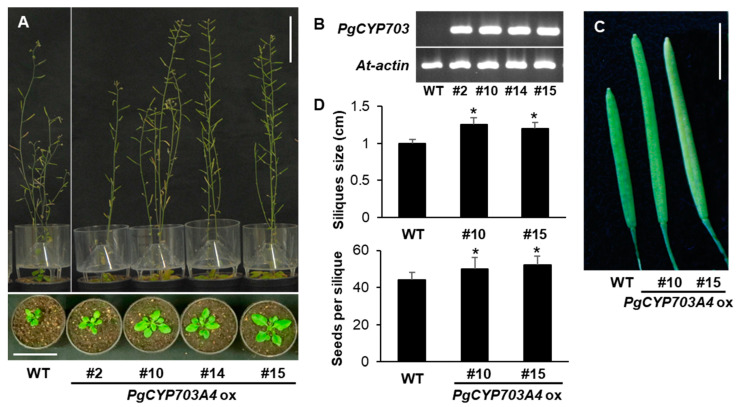
Phenotype analysis of PgCYP703A4 overexpressing A. thaliana. (**A**) Growth phenotype of four different PgCYP703A4 overexpression lines and wild type at 2 week- and 7 week- old. Scale bar indicates 5 cm. (**B**) Detection of PgCYP703A4 transcription in transgenic A. thaliana’s rosette leaves. At actin served as the control. (**C**,**D**) Silique size of PgCYP703A4 overexpression lines. Scale bar indicates 5 mm. Values indicate mean of 20 biological replicates ± SD. * *p* < 0.05.

Due to difficulties in obtaining transgenic regenerated *P. ginseng* plants, we generated *PgCYP703A4* overexpressing transgenic Arabidopsis (*PgCYP703A4ox*) to examine its functional role in planta (Figure 4A–D). The stable incorporation of the *PgCYP703A4* gene and its heteroexpression was confirmed via RT-PCR (Figure 4B). *PgCYP703A4ox* produced slightly taller plants compared with the wild type, but not significant (Figure 4A). Notably, the siliques increased in size by 20% compared with the wild type and *PgCYP703A4ox* siliuqes contain higher number of seeds than wile type significantly (Figure 4C,D). 

*A.thaliana CYP703A3* mutant was reported to show impaired pollen walls lacking a normal exine layer, which leads to partial male sterility [8]. To determine how *PgCYP703A4* affects pollen wall formation, we observed the anthers and pollen phenotype by semi-thin cross-section and SEM (Figure S3). The anther, pollen, and pistil of *PgCYP703A4ox* appeared similar to the wild type, whereas *CYP703A3* exhibited aborted pollen without outer elegant wall formation (Figure S3). Therefore, pollen viability and reproductive organ function was not altered by *PgCYP703A4* gene overexpression. 

### 3.5. Fatty Acid Composition of PgCYP703A4-Overexpressing A. thaliana

The cuticle, a hydrophobic layer that coats the surface of the aerial organs, such as leaves, stems, flowers, and fruits, is a biopolymer that is composed of two classes of lipophilic constituents, namely, cutin and waxes [35,36]. Since the silique phenotype of *PgCYP703A4ox* is elongated and the exocarp is made of cutin, we further conducted GC-MS and GC-FID.

**Figure 5:**
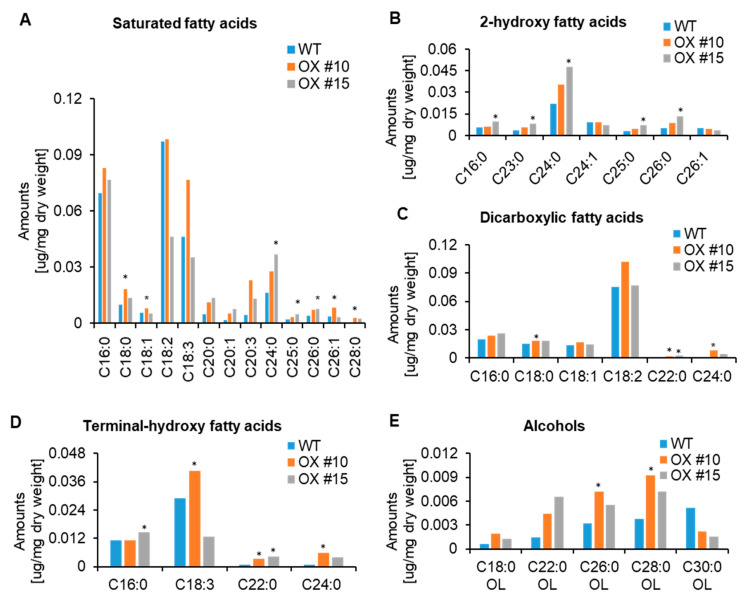
Chemical analysis of silique cutin monomers in wild type and PgCYP703ox lines via GC-MS and GC-FID. (**A**) Saturated fatty acids per milligram of dry weight (µg/mg). (**B**) 2-hydroxy fatty acids per milligram of dry weight (ug/mg). (**C**) Dicarboxylic fatty acids per milligram of dry weight (ug/mg). (**D**) Terminal-hydroxy fatty acids per milligram of dry weight (ug/mg). (**E**) Alcohols per milligram of dry weight (ug/mg). Values indicate mean of five biological replicates ± SD. * *p* < 0.05.

The overall sum of cutin monomers were not significantly different between wild type and *PgCYP703ox* lines. We found that two independent lines alter the composition of fatty acids, with some variation, which might explain complex fatty acids metabolism for the cutin polymer. For example, compared with the wild type, the *PgCYP703A4ox* #10 significantly increased saturated fatty acids of C18:0, C18:1, C26:1 and C28:0, (Figure 5A), dicarboxylic fatty acids of C18:0, C22:0, C24:0, and C26:0 (Figure 5C) and terminal-hydroxy fatty acids of C18:3, C22:0, and C24:0 (Figure 5D), and C28:0 alcohol type (Figure 5E). *PgCYP703A4ox* #15 increased C24:0, C25:0, and C26:0 saturated fatty acids (Figure 5A), and 2-hydroxyl fatty acids, such as C16:0, C23:0, C24:0, C25:0, C26:0 (Figure 5B). The dicarboxylic fatty acids of C22:0 and C24:0 were significantly increased in the *PgCYP703A4ox* lines (Figure 5C). The levels of the terminal-hydroxy fatty acid C22:0 was found to increase up to two times in *PgCYP703A4ox* in comparison with the wild type (Figure 5D). These data indicate that the *PgCYP703A4* overexpression in *A*. *thaliana* affects the fatty acid composition of cutin monomers in siliques.

## 4. Discussion

Plants have evolved a variety of enzymes for the in-chain α-, β-, and ω-hydroxylation of fatty acids. Hydroxylated fatty acids are the biosynthetic intermediates of plant biopolymers, such as cutin and suberin, which make up the barriers from land plant stress situations. Thus, fatty acid metabolic enzymes are critical for plants; however, the role of CYP members in controlling the development of *P. ginseng* has not been well studied. In this study, we characterized *PgCYP703A4* and its role in the fatty acid metabolism. The *PgCYP703A4* expression only detected at the flowering stage during microspore formation in the tapetum and gamete cells, which are active in sporopollenin synthesis. The overexpression of *PgCYP703A4* in *A. thaliana* increased silique size and seed production without affecting gamete cell, similar to *PgCYP704B1* [15].

CYPs constitute the largest family of enzymes in plant metabolism and represent plant evolution in terms of plant metabolism in development and adaptation, such as signaling, defense, and polymerization of complex chemical substances [37]. Among the 11 land plant clans, the CYP71 clan represents more than half of all CYPs in higher plants; consequently, a wide diversity of functions makes them more difficult to predict their preferred substrates than other clans [37]. In addition to CYP703, CYP77 family members, AtCYP77A4 and AtCYP77A6, can in-chain-hydroxylate fatty acids to form precursors of cutin [9,38,39]. Looking at their phylogenetic relationship, CYP703 diversified prior to the emergence of CYP77s as spore protectors. CYP703 is an ancient gene family that is required for land plants, whereas CYP77 is required in only angiosperms [37]. However, both CYP703A and CYP77A function as in-chain hydroxylases, compared with most other enzymes that are end-chain (ɷ) hydroxylase [30].

In addition, a part of the CYP71 clan, the CYP78 subfamily genes exhibited fatty acid hydroxylation reactions, particularly for short chains [24]. Different from a single member of CYP703 (Figure 2), the CYP78 family contains several members in *A. thaliana* (Figure 2), indicating late gene duplication for the species. CYP78A family members regulate reproductive organ development but are more related to female organs. *A. thaliana* gene *CYP78A9* was reported to be involved in the control of carpel shape [40]. The overexpression of *CYP78A9* results in large, seedless fruit, although the metabolites have not been discovered [40]. *O.* *sativa* gene *OsCYP78A13* promotes seed growth by regulating the embryo and endosperm size, as well as spikelet hull development [41]. *PaCYP78A9* regulates fruit size in *Prunus avium*, showing increases in silique and seed size in *A. thaliana* by hetero-overexpression [42]. In addition to the above studies of the CYP71 clan other functions include glucosinolate production [43], p-coumaraldehyde hydroxylation [44], and pathogen defense function [45], as indicated in Figure 2. It is clear that the CYP71 clan has a large diversity of functions, but only CYP703 and CYP78 families of this clan, have the conserved PERF consensus, and both subfamiles are involved in plant reproductive development. Further studies are required to identify its positive relationship with biological function.

In *P.* *ginseng*, reproductive development and functional studies are scarce. We previously identified a functional ortholog of *AtCYP704B1*, termed *PgCYP704B1* [15]. The CYP704B family, which belongs to the CYP86 clan, is involved in the ω-hydroxylation of long-chain fatty acids. Altered exine in the pollen wall was detected in mutant of *A. thaliana cyp704B1* [46], *Brassica napus CYP704B1* [47], and *O. sativa CYP704B2* [36]. However, *O.* *sativa CYP704B2* also had an undeveloped anther cuticle and sterile male phenotype [36]. It is similar to CYP703A, although it is in a separate clan. Similarly, CYP701A and CYP88A, which belong to the CYP71 and CYP85 clans, respectively, act sequentially in the same pathway as ent-kaurene oxidase and kaurenolic acid oxidase, respectively [48]. With the early evolution of CYPs, CYP703 and CYP704 could be involved in cutin biopolymer synthesis, particularly for pollen wall polymers, for in-chain and ɷ-hydroxylation, respectively. This study was limited by the difficulty of obtaining flowers from transgenic *P.* *ginseng*. However, further studies on *P.* *ginseng* development is required to develop hybrid and male sterile system for breeding.

Taken together, *PgCYP703A4*, a member of CYP703A in the CYP71 clan, and *PgCYP704B1* [22], in the CYP86 clan, are similarly expressed in the *P. ginseng* tapetum and meiotic cells, and overexpression in *A. thaliana* affects fatty acid metabolism in siliques. Previous studies on *A. thaliana* and *O. sativa* examined knockout mutants displaying partial or full male sterility [7,9,36,46] and therefore did not further investigate the phenotype regarding fruit development. This requires further investigation to determine the role of hydroxylated fatty acids in sporopollenin synthesis and the development of the silique cuticle.

## Data Availability

Not applicable.

## Supplementary Materials

The following are available online at https://www.mdpi.com/article/10.3390/plants11030383/s1, Figure S1: Multiple alignment of PgCYP703 with homologous proteins from other plant species. Figure S2: Superimposed hydrophobicity profile predictions for PgCYP703A4 and selected homologs from the 703A4 family. Figure S3: Cytological analysis of an *Arabidopsis* mutant and the *PgCYP703A4* overexpression line.
