# Supplementary material for: Overexpression of the Panax ginseng CYP703 Alters Cutin Composition of Reproductive Tissues in Arabidopsis
Source: Plants (Basel). 2022 Jan 30;11(3):383. doi: 10.3390/plants11030383 (PMC8839735; doi:10.3390/plants11030383)
Supplement: Supplementary file 1 [file plants-11-00383-s001.zip › plants-1547635-supplementary.pdf]

Supplementary Data

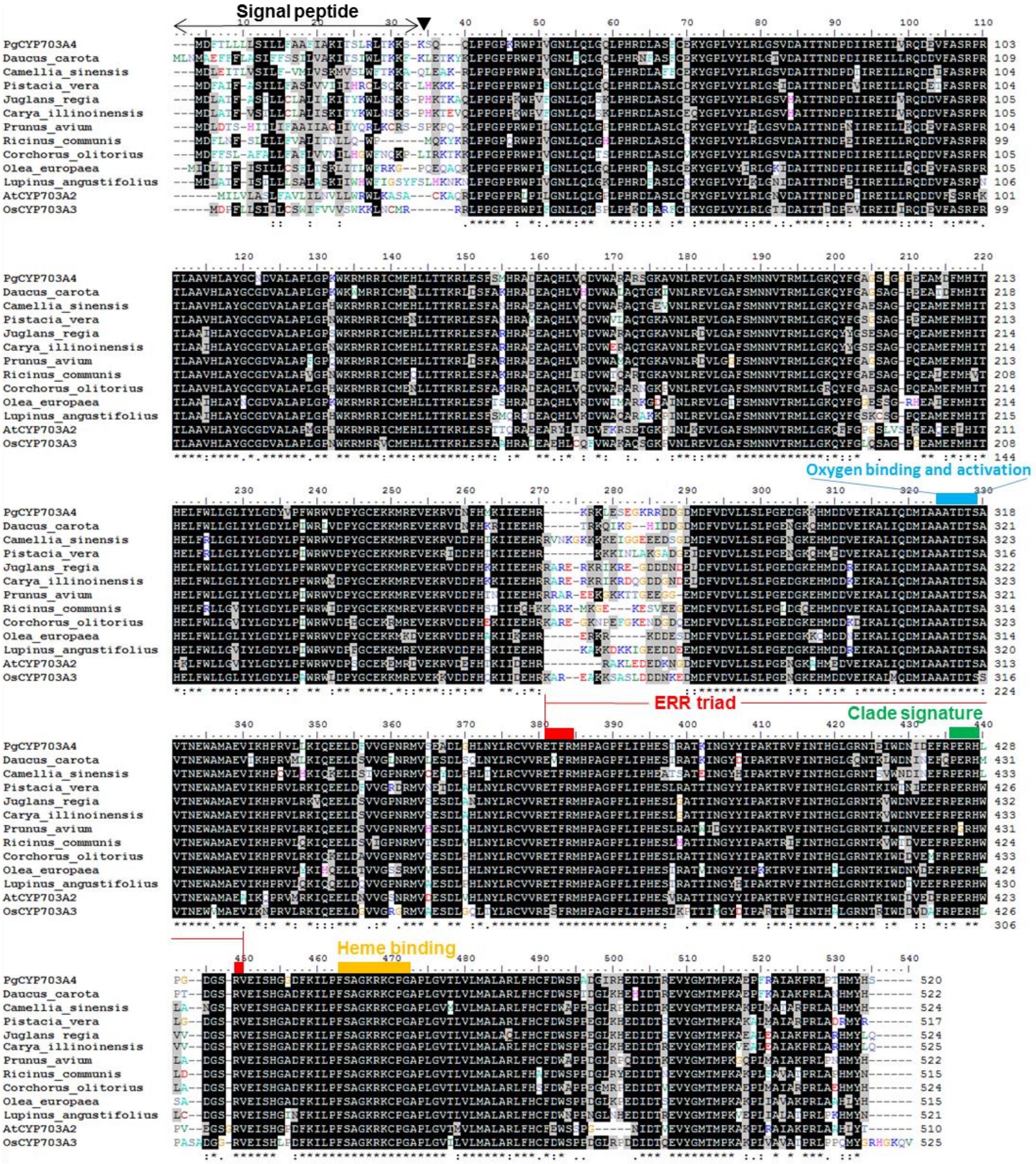

Figure S1. Multiple alignment of PgCYP703 with homologous proteins from other plant species. *Camellia sinensis* (XP 028126426.1), *Prunus avium* (XP 021815068.1), *Lupinus angustifolius* (XP 019465190.1), *Olea europaea* (CAA3000330.1), *Juglans regia* (XP 018818345.1), *Pistacia vera* (XP 031263346.1), *Daucus carota* (XP 017234282.1), *Panax ginseng* (PgCYP703A4), *Arabidopsis thaliana* (At1g01280), and *Oryza sativa* (Os08g0131100). Identical and similar residues are shown on black and gray backgrounds, respectively. The gaps required for optimal alignment are indicated by dashes. Red squares show conserved sequences of P450 genes.

Conserved characteristics of CYP450 proteins [29] and the clade signature of CYP703 subfamilies are indicated.

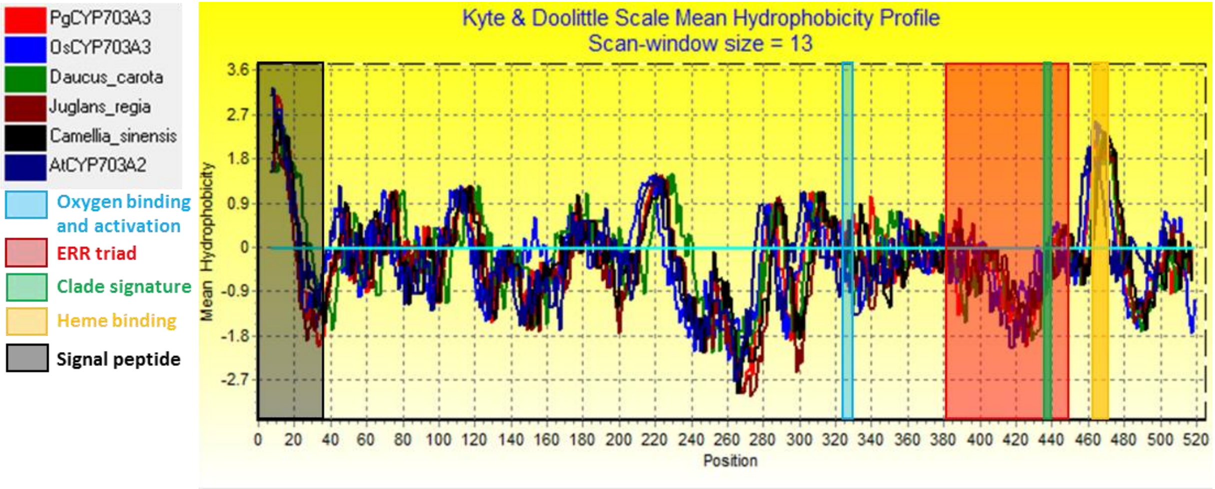

Figure S2. Superimposed hydrophobicity profile predictions for PgCYP703A4 and selected homologs from the 703A4 family. Hydrophobic domains and hydrophilic domains are indicated by positive numbers and negative numbers, respectively. Conserved motifs corresponding are depicted as in Supplementary Figure 1.

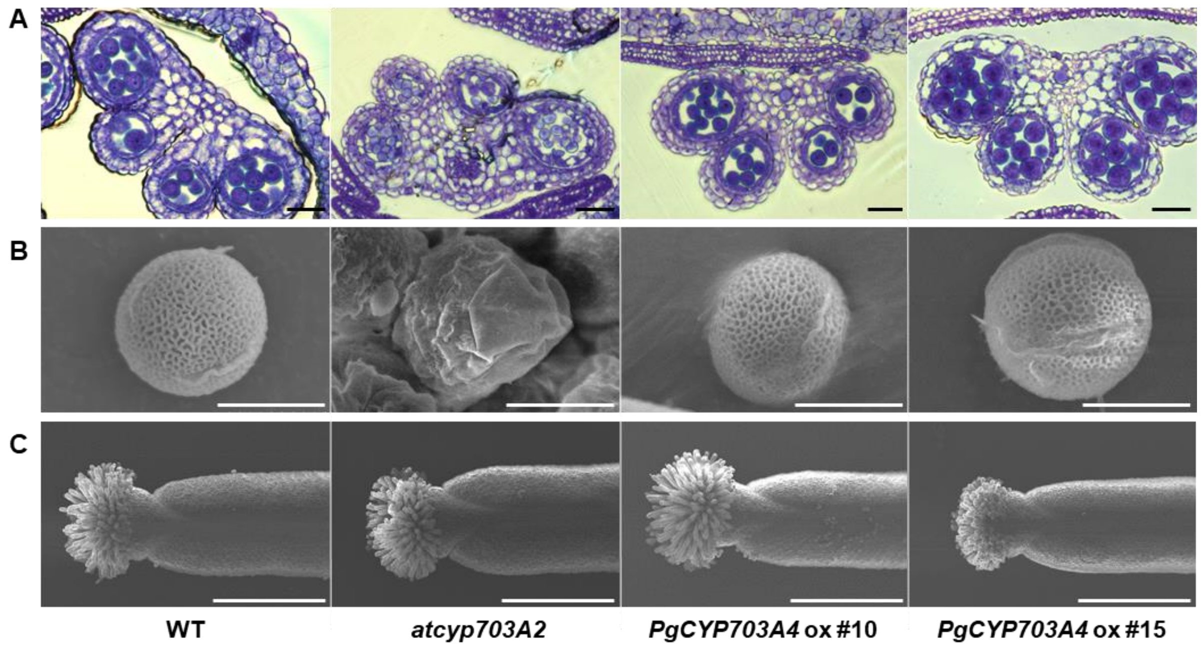

Figure S3. Cytological analysis of an *Arabidopsis* mutant and the *PgCYP703A4* overexpression line. (A) Semi-thin section of mature anther of wild type (WT), *AtCYP703A2* mutant (*atcyp703A2*), and two independent *PgCYP703A4*-overexpressing lines (*PgCYP703A4* #10 and *PgCYP703A4* #15) Arabidopsis. Scale bars indicate 200 μm. (B and C) Scanning electron microscopic analysis of mature pollen (B) and pistil (C) of each line's flower. Scale bars indicate 10 μm for (B) and 400 μm for (C).
